# Cost-Effectiveness Analysis of Axicabtagene Ciloleucel vs. Tisagenlecleucel for the Management of Relapsed/Refractory Diffuse Large B-Cell Lymphoma in Spain

**DOI:** 10.3390/cancers14030538

**Published:** 2022-01-21

**Authors:** Mariana Bastos-Oreiro, Ana de las Heras, María Presa, Miguel A. Casado, Carlos Pardo, Victoria Martín-Escudero, Anna Sureda

**Affiliations:** 1 Hematology Department, Gregorio Marañón Health Institute, 28007 Madrid, Spain; bastosmariana@yahoo.com; 2 Health Economics Department, Pharmacoeconomics & Outcomes Research Iberia (PORIB), 28224 Madrid, Spain; mpresa@porib.com (M.P.); ma_casado@porib.com (M.A.C.); 3 Market Access, Reimbursement & Health Economics and Outcomes Research Department, Gilead Sciences, 28033 Madrid, Spain; carlos.pardo@gilead.com (C.P.); victoria.martinescudero@gilead.com (V.M.-E.); 4 Hematology Department, Institut Català d’Oncologia-Hospitalet, IDIBELL, Universitat de Barcelona, 08908 Barcelona, Spain; asureda@iconcologia.net

**Keywords:** axicabtagene ciloleucel, tisagenlecleucel, diffuse large B-cell lymphoma, cost-effectiveness analysis, cost-utility analysis

## Abstract

**Simple Summary:**

CAR T therapies axicabtagene ciloleucel (axi-cel) and tisagenlecleucel (tisa-cel) have been approved in Spain for patients with relapsed/refractory (R/R) diffuse large B-cell lymphoma (DLBCL), given their favourable outcomes for overall survival and progression-free survival. However, the cost of these treatments must be weighed within the context of the Spanish health system. In this study, we assessed the cost-effectiveness and cost-utility of axi-cel vs. tisa-cel from the Spanish National Health System perspective. Using commonly applied willingness-to-pay thresholds in Spain, our analysis shows that axi-cel is highly cost-effective when compared to tisa-cel in R/R DLBCL. These results could be used to support decision-making criteria for axi-cel financing.

**Abstract:**

The study aimed to assess the cost-effectiveness of axicabtagene ciloleucel (axi-cel) vs. tisagenlecleucel (tisa-cel) for the treatment of relapsed/refractory (R/R) diffuse large B-cell lymphoma (DLBCL) after ≥2 lines of systemic therapy in Spain. A lifetime partitioned survival mixture cure model, which comprises pre-progression, post-progression, and death health states, was used to estimate the accumulated costs and outcomes in terms of life years gained (LYG) and quality-adjusted life years (QALY). A matching-adjusted indirect comparison was used to reweight patient-level data from ZUMA-1, the pivotal clinical trial for axi-cel, to aggregate-level data from the pivotal tisa-cel trial, JULIET. The analysis was performed from the National Health System perspective, thus only direct costs were included. Sensitivity analyses (SA) were performed. Axi-cel yielded 2.74 incremental LYG and 2.31 additional QALY gained per patient compared to tisa-cel. Total incremental lifetime costs for axi-cel versus tisa-cel were €30,135/patient. The incremental cost-effectiveness ratio of axi-cel versus tisa-cel resulted in €10,999/LYG and the incremental cost-utility ratio in €13,049/QALY gained. SA proved robustness of the results. Considering the frequently assumed willingness-to-pay thresholds in Spain (€22,000/QALY and €60,000/QALY), axi-cel is a cost-effective treatment vs. tisa-cel for adult patients with R/R DLBCL in Spain.

## 1. Introduction

Non-Hodgkin lymphoma (NHL) comprises a heterogenous group of lymphoproliferative malignancies originating primarily in B lymphocytes [1]. Aggressive subtypes of B-cell NHL include diffuse large B-cell lymphoma (DLBCL) and primary mediastinal B-cell lymphoma [1]. DLBCL is the most common subtype of B-cell NHL, accounting for approximately 30% of newly diagnosed cases of NHL, with an estimated incidence of 3.8/100,000 per year in Europe [2,3].

Despite the improvements in overall survival (OS) with the addition of rituximab, approximately 30% of patients with DLBCL will develop relapsed/refractory (R/R) disease after first line treatment, which remains a major cause of morbidity and mortality [2,4]. Patients with R/R DLBCL are frequently ineligible for stem cell transplant (SCT) and have poor OS, with an expected median OS of approximately 6 months with a 2-year OS of 20% [5].

In recent years, the treatment landscape has experienced a milestone with the development of chimeric antigen receptor (CAR) T-cell therapies, which have demonstrated not only remarkable improvements in OS, but also the possibility of achieving above 50% complete response (CR) rates for DLBCL patients [6]. Currently, there are two CAR T-cell therapies authorised in Spain for adults with R/R DLBCL: axicabtagene ciloleucel (axi-cel; Yescarta) and tisagenlecleucel (tisa-cel; Kymriah). In the ZUMA-1 trial, after 27 months follow-up, axi-cel showed an objective response rate (ORR) of 83% and CR of 58%, durable responses and a median OS greater than 2 years in patients with R/R large B-cell lymphoma (LBCL) [7]. In the JULIET trial, tisa-cel presented a best overall response rate of 52%: 40% showed CR and 12% partial response (PR), at a median follow-up of 14 months among patients with R/R DLBCL [8]. More recently, Oluwole et al. performed a matching-adjusted indirect comparison (MAIC) to compare outcomes across the patient populations of the ZUMA-1 and JULIET trials [9].

CAR T therapies have demonstrated promising outcomes in terms of efficacy; however, the cost has prompted health systems to identify ways to reduce budget impact. Consequently, several European countries have adopted different risk-sharing reimbursement strategies, in order to ensure their health systems’ sustainability and reduce the risk related to the uncertainty resulting from the limited real-world evidence available [10].

In view of the above, the purpose of the present study was to assess the cost-effectiveness and the cost-utility of axi-cel versus tisa-cel in the treatment of adult patients with R/R DLBCL after two or more lines of systemic therapy from the Spanish National Health System (NHS) perspective, using a MAIC to make the ZUMA-1 data for axi-cel more comparable to the JULIET data for tisa-cel.

## 2. Results

### 2.1. Base Case

In the base case scenario, over a lifetime horizon, axi-cel yielded 9.45 life years gained (LYG) and 7.47 quality-adjusted life years (QALY) per patient versus 6.71 LYG and 5.16 QALY per patient with tisa-cel, equivalent to 2.74 incremental LYG and 2.31 incremental QALY (Table 1). Table 1 shows the proportion of patients alive with each therapy according to different timepoints, from 1 to 40 years.

Regarding the total expenditure over a lifetime horizon, axi-cel represented a total cost of €430,747 per patient compared with €400,612 with tisa-cel, which corresponds to an incremental cost of €30,135 (Table 1). Costs were somewhat higher for axi-cel compared with tisa-cel, particularly the pre-progression disease management costs, due to a longer progression-free survival (PFS) with axi-cel (incremental costs €24.561), as well as the drug acquisition costs (incremental costs €6720) and SCT costs (incremental costs €1327). Conversely, patients treated with axi-cel incurred lower costs in the post-progression state (incremental costs −€2268) (Table 1).

This resulted in an incremental cost-effectiveness ratio (ICER) of €10,999/LYG and an incremental cost-utility ratio (ICUR) of €13,049/QALY gained with axi-cel in comparison to tisa-cel (Table 1), suggesting that axi-cel may be a cost-effective treatment versus tisa-cel in the treatment of R/R DLBCL in Spain, at frequently used willingness to pay (WTP) thresholds of €22,000/QALY [11] and €60,000/QALY [12].

### 2.2. Sensitivity Analysis

**Figure 1:**
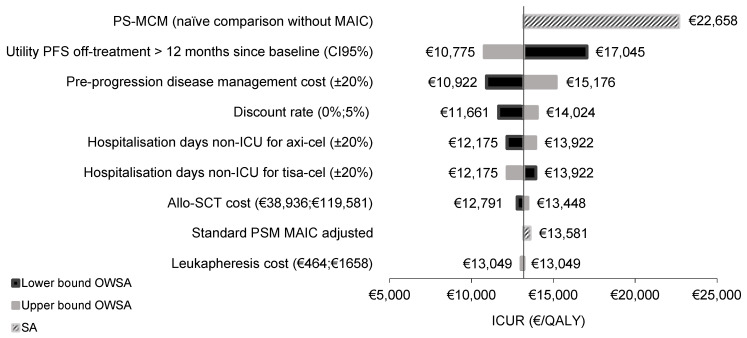
One-way sensitivity analysis and scenario analysis tornado diagram.

The results of the one-way sensitivity analysis (OWSA) demonstrated the model’s robustness. The most influential parameters were utility values in the PFS state >12 months after baseline and cost of disease management during this same state. Regarding the scenario analyses (SA), the naïve analysis (without MAIC adjustment) resulted in €22,658/QALY (Figure 1).

**Figure 2:**
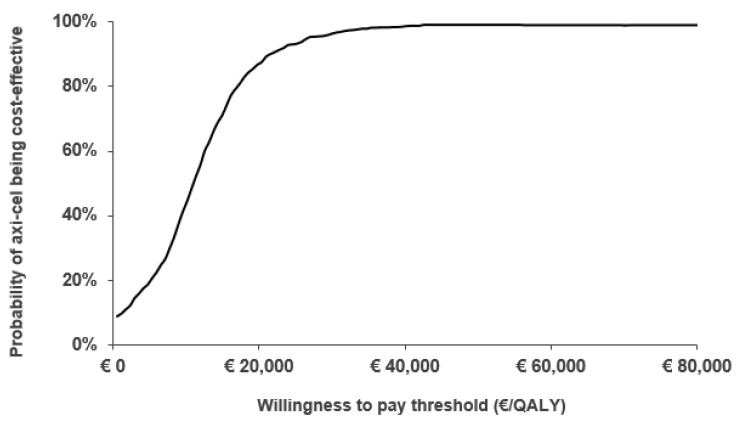
Cost-effectiveness acceptability curve of axi-cel vs. tisa-cel. Axi-cel, axicabtagene ciloleucel; QALY, quality-adjusted life year; Tisa-cel, tisagenlecleucel.

A cost-effectiveness acceptability curve (Figure 2) was used to show the probabilistic sensitivity analysis (PSA) results. Out of 1000 Monte-Carlo simulations performed, 92.3% and 99.2% of the cases presented an ICUR under €22,000/QALY and €60,000/QALY gained willingness-to-pay thresholds, respectively [11,12].

Allo-SCT, allogenic stem cell transplant; Axi-cel, axicabtagene ciloleucel; ICU, intensive care unit; ICUR, incremental cost-utility ratio; MAIC, matching adjusted indirect comparison; OWSA, one-way sensitivity analysis; PFS, progression-free survival; PSM, partitioned survival model; PS-MCM, partitioned survival mixture cure model; QALY, quality-adjusted life year; SA, scenario analysis.

## 3. Discussion

Prior to the arrival of CAR T-cell therapies, the prognosis for patients with R/R DLBCL was very poor [5] with few treatment options, constituting an urgent unmet need for effective treatments [2]. Nevertheless, CAR T-cell therapies have changed the treatment paradigm, demonstrating promising survival outcomes in the treatment of these patients. Further follow-up from the pivotal trials in addition to real-world evidence is warranted to confirm the effectiveness of these therapies. For this reason, the Spanish NHS has launched a plan for advanced therapies, including CAR T-cell therapies, known as the “*Plan para el abordaje de las terapias avanzadas en el SNS: medicamentos CAR*”, with the objective of guaranteeing the efficient and safe use of these innovative treatments. The latest of these reports published in December 2020 by the Spanish NHS, confirms these preliminary outcomes in Spanish hospitals, in terms of efficacy and safety in a real-world environment [13].

Our study is the first to compare the efficiency of the two authorised CAR T-cell therapies in the treatment of R/R DLBCL from the Spanish NHS perspective. When comparing axi-cel versus tisa-cel, the analysis showed an increment of 2.31 QALY per patient and incremental costs of €30,135 per patient, which leads to an ICUR of €13,049/QALY gained. This value stands below the frequently cited WTP thresholds of €22,000–€60,000 per QALY gained [11,12], used to establish whether the therapy of interest represents an efficient use of limited NHS resources in the Spanish setting.

Recently, a cost-utility analysis developed from the USA payer perspective for the same comparators presented axi-cel as a dominant alternative vs. tisa-cel, resulting in higher QALY gained (+2.31 discounted) and slightly lower costs (−$1407) [14]. QALY gained for axi-cel vs. tisa-cel were equal from USA and Spanish payer perspectives, however total costs were higher for tisa-cel in the USA analysis, in contrast with our analysis where axi-cel showed higher costs. Additionally, the efficiency of axi-cel and tisa-cel has been evaluated separately, in comparison to chemotherapy schemes in the DLBCL context in different countries. In Europe, Marchetti et al. assessed the efficiency of axi-cel versus best supportive chemotherapy from the Italian health system perspective, resulting in an ICUR of €44,746/QALY gained [15]. From the USA payer perspective, Roth et al. revealed an ICUR of $55,128/QALY gained when they compared axi-cel with salvage chemotherapy [16]. Regarding tisa-cel, few economic evaluations for DLBCL have been identified. An analysis from the Canadian payer perspective yielded an ICUR of $103,112/QALY gained versus salvage chemotherapy [17], very similar to the CHF113,179/QALY (1 CHF = 0.92 €) obtained in an analogue analysis from the Swiss healthcare system perspective [18].

From an economic point of view, several European countries have adopted risk-sharing financial schemes consisting of staged payments based on collected clinical outcomes, resulting in pioneering reimbursement strategies within the European Union (EU) [10]. In particular, the Spanish Ministry of Health (MoH) has developed a tool known as VALTERMED (*Valor Terapéutico en la Práctica Clínica Real de los Medicamentos de Alto Impacto Sanitario y Económico en el Sistema Nacional de Salud*), where data generated in clinical practice are collected, in order to determine the real value of therapies [19]. Other countries such as France and England, with the aim of accelerating patient access to these therapies, have accepted conditioned rebates that will be re-assessed upon longer follow-up results of the clinical trials and upcoming real-world data. Given the current scenarios, CAR T-cell therapies are not only cutting-edge treatments in terms of clinical efficacy, but also with regard to a liable method of payment that achieves an optimal “value for money”.

In the absence of any head-to-head trial comparing axi-cel and tisa-cel, outcomes were extracted from the MAIC recently published by Oluwole et al., applying matched ZUMA-1 trial individual patient data (IPD) to aggregate-level data from the JULIET trial in order to adjust for cross-trial differences [9]. The MAIC approach is consistent with the recommendations gathered in the National Institute for Health and Care Excellence (NICE) Decision Support Unit Technical Support (DSUTS) Document 18 [20], that consider the MAIC as a valid option when IPD is available for one trial and aggregate data are available for the other trial. The main advantages of the MAIC consist of its solid literature-based content versus a simulated treatment comparison, its straightforward interpretation, and importantly, its recognition among Health Technology Assessment (HTA) agencies [9].

With regard to the survival estimates used in the analysis, a fitted partitioned survival mixture cure model (PS-MCM) was applied for the entire model time horizon, given its superior robustness in contrast with a piecewise approach. The piecewise approximation utilises the observed survival in the form of Kaplan-Meier (KM) curves followed by the PS-MCM fits for the post-trial period, carrying a high uncertainty around the survival estimates towards the end of the observed time, due to a low number of patients still at risk. On the other hand, the PS-MCM approach is widely accepted by HTA agencies. In fact, NICE submissions for axi-cel and tisa-cel in R/R LBCL adopted the PS-MCM approach [21,22], as well as previous USA cost-effectiveness for axi-cel in R/R LBCL [16,23].

The current analysis has some limitations. Firstly, the lack of a direct comparison of axi-cel against tisa-cel in a head-to-head clinical trial can be considered as a potential drawback of the analysis. By means of a MAIC approach, clinically relevant cross-trial differences have been balanced; however, there are some co-variates that could not be adjusted and could be interpreted as a source of bias [9,24]. For instance, use of bridging chemotherapy was allowed in JULIET trial but not for ZUMA-1 patients, making it impossible to evaluate the impact of this parameter. Furthermore, there were certain disparities in the scheduling process and time from leukapheresis to infusion that were not adjusted: in ZUMA-1, patients would undergo leukapheresis only if a manufacturing slot was available, whereas in JULIET, patients were subject to leukapheresis, with independence of slot availability. Additionally, definition of “relapsed disease” at baseline was unequal between ZUMA-1 and JULIET, admitting a lower-risk population in JULIET by including all patients that experienced progression at any time after two lines of therapy, in comparison to ZUMA-1, where patients presented progressive or stable disease as a best response to the last line of therapy, or had relapsed within 12 months after SCT [7,8]. In order to account for the possible impact of the MAIC technique on the analysis, a SA without considering the MAIC (naïve comparison) for survival outcomes was performed.

Furthermore, PFS and OS data for axi-cel and tisa-cel are limited to a median follow-up of 27 and 14 months respectively; thus, parametric survival distributions were required to extrapolate trial KM curves. The extrapolated survival data could not represent real life. However, the modelling approach used in this analysis is consistent with prior studies, which suggest that mixture cure models can provide a more accurate estimate of long-term survival compared with standard models when a substantial fraction of the modelling population achieve long-term remission [25,26,27]. Despite the extrapolation approach, survival data from patients treated with both therapies should continue to be collected to reduce the uncertainty in long-term survival.

Another limitation present in the study lies in the fact that cytokine release syndrome (CRS) and neurological events were the only grade ≥3 adverse events (AEs) considered in the analysis, in line with the considerations of the MAIC publication [9]. Other possibly relevant AEs, such as cytopenias or infections, were excluded from the analysis. Furthermore, no disutility values were attributed to AEs similar to other economic evaluations developed for axi-cel [14,16].

Despite the limitations described previously, the results of the sensitivity analyses confirmed that the uncertainty associated with the parameters used in the modelling did not represent a significant deviation from the results obtained in the base case, except for the naïve comparison without MAIC, which turned out to be the most influential variable, still remaining cost-effective when applying the €60,000/QALY gained threshold. The average ICER derived from the PSA was €11,114/QALY gained, which is congruent with the deterministic ICER of €13,049/QALY estimated in the base case, indicating a limited uncertainty surrounding model parameters and assumptions.

## 4. Materials and Methods

### 4.1. Model Structure

**Figure 3:**
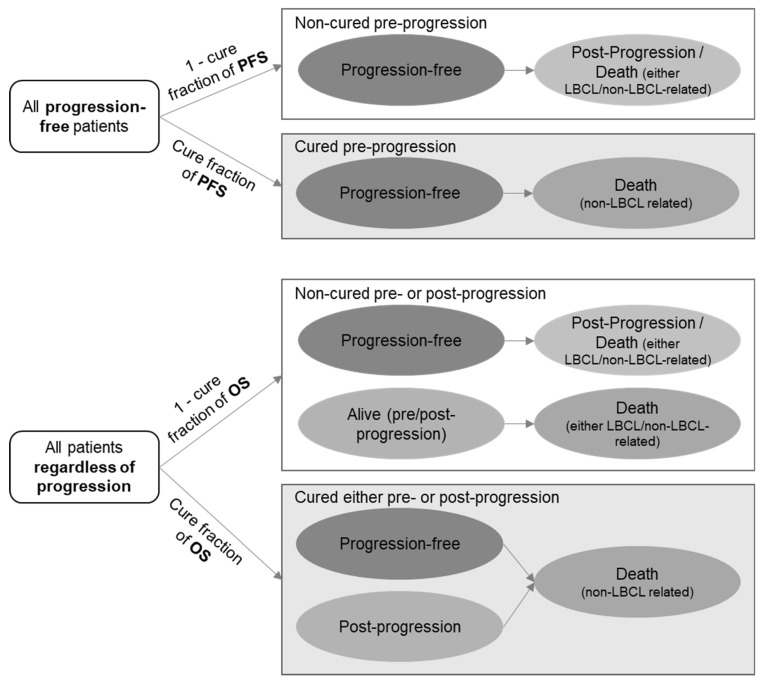
Decision-analytic model: Partitioned survival mixture cure model for axi-cel and tisa-cel. LBCL, large B-cell lymphoma; OS, overall survival; PFS, progression-free survival.

A previously developed decision-analytic model in Microsoft Excel^®^ (Excel 365, Microsoft, Redmond, WA, USA) was adapted to estimate over a lifetime horizon (maximum of 50 years) the costs and health benefits, in terms of LYG and QALY, accrued for a hypothetical cohort of adult patients with R/R DLBCL after ≥2 lines of systemic therapy who received axi-cel or tisa-cel [14]. According to the approach commonly used in advanced stage oncology models, a PS-MCM was utilised for both treatment arms. The model comprised pre-progression, post-progression and death health states, to represent the long-term survival observed on the 27-month follow-up ZUMA-1 trial [7] and 14-month follow-up JULIET trial [8]. Survival curves in these trials may acquire an “L-shape” with a “long tail” or plateau, which originates from a proportion of patients that can experience long-term remission or cure. When applying a PS-MCM for PFS and OS, patients in these states are divided into two groups: non-cured patients at risk of disease-related progression or death, as well as general mortality (due to non-DLBCL causes), and cured patients, only exposed to general mortality (will not experience disease-related progression or death). Transitions between health states were conducted in one-month cycles to accurately capture the time resolution of disease progressions and survival outcomes (Figure 3), and were half-cycle corrected.

The analysis was carried out from the perspective of the Spanish NHS and a discount rate of 3% was applied to costs and health outcomes, in accordance with the published recommendations for economic evaluations in Spain [28].

All the parameters included in the present analysis were reviewed and validated by two experts in the haemato-oncology field.

### 4.2. Population

The patient population included in the analysis were adult patients with R/R DLBCL, adjusting ZUMA-1 (pivotal trial of axi-cel) patient population to JULIET (pivotal trial of tisa-cel) population through the MAIC [9]. Average patient age was 58 years-old with a 64% proportion of men, in line with the most recent report published by the Spanish MoH related to the follow-up of advanced therapies [13]. An average weight of 70 kg and average body surface of 1.70 m^2^ were assumed, based on typical characteristics of the Spanish population [29].

### 4.3. Treatment Strategies

The alternatives compared in the analysis included a single infusion of axi-cel versus a single infusion of tisa-cel. For both CAR T therapies, the model tracked patients from treatment initiation, considering the leukapheresis procedure to harvest T-cell and the treatment with lymphodepleting chemotherapy, which differed between treatments. For axi-cel, patients received cyclophosphamide 500 mg/m^2^ and fludarabine 30 mg/m^2^ intravenously on the 5th, 4th, and 3rd day before infusion [7], while tisa-cel required cyclophosphamide 250 mg/m^2^ and fludarabine 25 mg/m^2^ intravenously daily for 3 days [8].

### 4.4. Clinical Data

To determine the distribution of patients across health states, PFS and OS trial curves were extrapolated to the lifetime horizon. For tisa-cel, OS and PFS data were obtained from JULIET [8], whereas clinical results for axi-cel were provided by ZUMA-1 [7]. A MAIC approach was applied in order to adjust for differences in patient characteristics between trials [9], where patient-level data from ZUMA-1 was adjusted to aggregate data of JULIET through a logistic regression model based on the propensity score, for the following key patient characteristics for its clinical relevance: International Prognostic Index (IPI) score (<2 or ≥2), Eastern Cooperative Oncology Group (ECOG) score (0/1), disease stage (<3 or 3/4), refractoriness (relapsed/refractory), double/triple hit status, cell of origin (DLBCL/other types of LBCL) and number of prior lines of therapy (<3 or 3/≥4).

To establish the proportion of patients in each health state (pre-progression state, post-progression state and death) over a lifetime horizon, definitive survival curves for both treatments were estimated as a weighted average of the survival curves of cured and non-cured subpopulations, according to their relative proportions. Axi-cel survival curves of the overall population were adjusted to a Gamma distribution for OS and a Log-logistic distribution for PFS. Regarding tisa-cel, overall population OS and PFS curves were fitted to Log-normal and Log-logistic distributions, respectively. For the cured group, survival curves were based on the mortality of the Spanish general population; thus, patients in this group could only move to death health state [30].

Additionally, the model considered CRS and neurological AEs grade ≥3 based on the MAIC approach [9]. To prevent double counting, no additional AEs were allocated, assuming these occurred while patients were hospitalized for treatment infusion.

### 4.5. Utilities

In order to estimate the QALY gained, utility values were associated with each health state based on EQ-5D data from a safety management cohort of ZUMA-1 [31] (Table 2). Different utility values were established depending on whether the patient was on or off-treatment and time since completion of treatment, regardless of the CAR T-cell therapy.

On the first month after treatment, a utility of 0.740 was assigned. After this first month, and if patients had not progressed, they could experience an improvement in their quality of life, thus increasing their utility value up to 0.782. According to the experts’ opinion, if no progression occurred in the 12 months since baseline, thus reaching long-term remission, a utility value of 0.820 was achieved. For the post-progression state, a utility value of 0.390 was designated, as stated by Chen et al. [32].

### 4.6. Resource Use and Costs

According to the Spanish NHS perspective, only direct healthcare costs were included, comprising axi-cel and tisa-cel acquisition costs, drug administration and monitoring, SCT, health state management, end-of-life care and AEs management costs. Axi-cel and tisa-cel acquisition costs were based on published ex-factory prices [33] with national mandatory deduction applied [34] (Table 3). CAR T administration and monitoring costs, applied equally for both therapies, involved hospitalisation in an intensive care unit (ICU) and non-ICU for 7 days and 14 days, respectively, laboratory tests, specialist visits and blood transfusions. Other resources included for both CAR T-cell therapy-related costs were: leukapheresis procedure, lymphodepleting chemotherapy drug acquisition and administration (including hospitalisation for 5 days, laboratory tests and antiemetic treatment) (Table 3).

Disease management costs by health states were estimated based on the disaggregated resource consumption referred to specialist visits, hospitalisation, diagnostic and laboratory tests and concomitant treatments, provided by the expert panel, equal for both treatment arms (Table 3). End-of-life care costs were also considered for both treatments, regardless of the cause of death.

Regarding SCT, 7.90% and 5.40% of patients who relapsed or were refractory to CAR T cell treatment in the axi-cel and tisa-cel arm, respectively, were assumed to undergo allogeneic transplantation (allo-SCT) as shown in the ZUMA-1 [7] and JULIET [8] data. ZUMA-1 did not report any autologous transplantation for axi-cel in contrast with 0.90% of patients receiving tisa-cel detailed by JULIET (Table 3).

Grade ≥3 AEs-related costs included the management of CRS and neurological events with tocilizumab (an anti–interleukin 6 receptor monoclonal antibody), in addition to imaging and laboratory tests and antibiotics, only for CRS (Table 3).

Drug acquisition costs of lymphodepleting chemotherapy, concomitant treatments and tocilizumab were estimated based on published ex-factory prices [33] applying the national mandatory deductions when applicable [34]. No vial wastage was considered in the cost calculations.

Unitary costs for the health resources were obtained from local national database of health costs [35]. All costs were expressed in euros, 2020 values, and for those costs obtained from literature, the cost was inflated in 2020 based on the Spanish general consumer price index [36].

### 4.7. Outcomes

Total costs, LYG and QALY gained were estimated for both therapies. The cost-effectiveness results, presented as ICER and ICUR, were expressed as incremental cost in euros per survival increase measured as LYG and QALY gained, respectively.

### 4.8. Sensitivity Analysis

OWSA, SA and PSA were performed to assess the robustness of the model and to test the uncertainty surrounding model parameters on the ICER and ICUR results.

For the OWSA, the following parameters were varied individually: discount rate (0% and 5%) as suggested by López-Bastida et al. [28], cost of allo-SCT (minimum [€38,936] and maximum [€119,581] values collected in the national database of health costs), cost of leukapheresis (minimum [€464] and maximum [€1658] values collected in the national database of health costs), non-ICU hospitalisation days for axi-cel and tisa-cel (±20%), cost of disease management in the pre-progression state (±20%), utility off-treatment >12 months since baseline in the pre-progression state (CI95%).

SA considering standard partitioned survival model (PSM), this is without considering a fraction of cured patients, and PS-MCM without alignment of patient characteristics through MAIC, were also tested. In the PSM scenario, a Gompertz distribution was applied for OS and PFS in both treatment arms. On the other hand, in the naïve comparison without MAIC, according to the best fitting based on Akaike’s information criterion (AIC), PFS for axi-cel and tisa-cel and OS for axi-cel were adjusted to a Log-logistic curve, while OS for tisa-cel was fitted to a Log-normal curve.

PSA was performed using 1000 Monte-Carlo simulations. The value of each key parameter would vary according to a specific probability distribution assigned to each parameter. Beta distributions were applied for utilities, AEs rates, dose intensity of lymphodepleting chemotherapy and AEs management treatments, proportion of patients hospitalized in ICU and non-ICU and proportion of patients undergoing SCT; and gamma distributions for all type of costs and hospital days.

## 5. Conclusions

Axi-cel demonstrated an increase in survival outcomes in terms of LYG and QALY gained compared with tisa-cel over a lifetime horizon, resulting in an ICER of €10,999/LYG and an ICUR of €13,049/QALY gained. These results show that axi-cel is a cost-effective therapy for the treatment of R/R DLBCL after two or more lines of systemic therapy in Spain, when using commonly used WTP thresholds in Spain. Sensitivity analyses demonstrated the consistency of the results, with PS-MCM without the MAIC adjustment being the most influential parameter, still, however, yielding an ICUR below the €60,000/QALY WTP threshold.

## Figures and Tables

**Table 1:** Base case results.

Health Outcomes	Axicabtagene Ciloleucel	Tisagenlecleucel	Incremental
**Overall survival**			
% alive at 1 year	75.3%	47.3%	28.0%
% alive at 2 years	61.2%	38.3%	22.8%
% alive at 5 years	50.1%	34.4%	15.7%
% alive at 10 years	45.2%	32.5%	12.7%
% alive at 20 years	36.8%	27.0%	9.8%
% alive at 30 years	19.8%	14.6%	5.2%
% alive at 40 years	2.2%	1.6%	0.6%
**Total LYG per patient**	**9.45**	**6.71**	**2.74**
LYG in pre-progression state	8.87	5.97	2.90
LYG in post-progression state	0.58	0.75	−0.16
**Total QALY per patient**	**7.47**	**5.16**	**2.31**
QALYs in pre-progression state	7.25	4.87	2.37
QALYs in post-progression state	0.23	0.29	−0.06
**Costs**			
**Total costs per patient**	**€430,746.52**	**€400,611.79**	**€30,134.73**
Drug acquisition costs ^†^	€313,920.00	€307,200.00	€6720.00
Leukapheresis costs	€1030.68	€1030.68	€0.00
Conditioning chemotherapy costs	€4270.89	€4234.24	€36.65
CAR T administration and monitoring costs ^‡^	€20,257.42	€20,257.42	€0.00
SCT costs	€5574.17	€4247.66	€1326.51
Health state medical costs: pre-progression state	€75,035.09	€50,473.64	€24,561.45
Health state medical costs: post-progression state	€8065.4	€10,333.30	−€2267.76
End-of-life care costs	€2299.85	€2548.29	−€248.44
AEs costs	€292.88	€286.56	€6.32
**ICER (axi-cel vs. tisa-cel)**		**€10,998.64/LYG**	
**ICUR (axi-cel vs. tisa-cel)**		**€13,048.58/QALY**	

^†^ Drug cost per administration. Axi-cel and tisa-cel drug costs were based on ex-factory list price applying a 4% mandatory deduction of Royal Decree Law 8/2010. ^‡^ CAR T administration and monitoring costs include ICU and non-ICU hospitalisation. AEs, adverse events; Axi-cel, axicabtagene ciloleucel; CAR T, chimeric antigen receptor T-cell; ICER, incremental cost-effectiveness ratio; ICUR, incremental cost-utility ratio; LYG, life years gained; QALY, quality-adjusted life years; SCT, stem cell transplant; tisa-cel: tisagenlecleucel.

**Table 2:** Utilities.

**Health State**	**Utility Value**
**Pre-progression** **[31]**	
Pre-progression: CAR T on treatment (1st month)	0.740
Pre-progression: off-treatment ≤12 months	0.782
Pre-progression: off-treatment >12 months	0.820
Post-progression [32]	0.390

CAR T, chimeric antigen receptor T-cell.

**Table 3:** Healthcare resource unitary costs (€, 2020).

**Treatment Related Costs**	**Cost**
Drug acquisition costs [33,34]	
Axi-cel: 0.4–2.0 × 10^8^ cells/kg	€313,920.00 ^†^
Tisa-cel: 1.2 × 10^6^−6.0 × 10^8^ cells/kg	€307,200.00 ^†^
Leukapheresis [35]	€1064.79
Lymphodepleting chemotherapy: axi-cel [33,34]	€174.84
Lymphodepleting chemotherapy: tisa-cel [33,34]	€138.18
CAR T administration and monitoring [35]	€8767.73
Allo-SCT [35]	€70,559.14
Auto-SCT [35]	€48,591.37
ICU hospitalisation (per day) [35]	€1338.45
Non-ICU hospitalisation (per day) [35]	€722.89
End-of-life care [35]	€3132.52
**Health states medical resource costs**	
Pre-progression health state (€/month) [33,34,35]	€704.89
Post-progression health state (€/month) [33,34,35]	€1153.43
**AE management cost**	
Cytokine release syndrome [33,34,35]	€1073.59
Neurological events [33,34,35]	€713.09

^†^ Drug cost per administration. Axi-cel and tisa-cel drug costs were based on ex-factory list price applying a 4% mandatory deduction of Royal Decree Law 8/2010. AE, adverse event; axi-cel, axicabtagene ciloleucel; CAR T, chimeric antigen receptor T-cell; CGCOF: Consejo General de Colegios Oficiales de Farmacéuticos (General General Council of the Association of Official Pharmacists database); ICU, intensive care unit; SCT, stem cell transplant; tisa-cel, tisagenlecleucel.

## Data Availability

The data presented in this study are available in this article.
